# Observations on the Neck as a Medical Region, on Trachelismus, and on Paroxysmal Diseases of the Nervous System

**Published:** 1849-10

**Authors:** Marshall Hall


### RECORD OF MEDICAL SCIENCE.

### PATHOLOGY AND PRACTICE OF MEDICINE.

*Observations on the Neck as a Medical Region, on Trachelismus, and on Paroxysmal Diseases of the Nervous System.* By MARSHALL HALL, M. D., F. R. S.—In my former paper I set forth the subject of what I ventured to designate TRACHELISMUS, and of its immediate consequence, PHLEBISMUS, as a principle of pathology at once newly detected, and of vast extent and importance. It embraces, indeed, more than three-fifths of the diseases of the nervous centres, as witnessed in private practice, constituting *the Class of Paroxysmal Diseases of the Nervous System,* one remaining fifth being inflammatory, and the other organic.

I now propose to proceed with my subject.

I will observe, in the first place, that the termination in *-itis* is become generic, and denotes the fact of the inflammatory character of disease.

I think a similar use may be made of the termination in *-ismus*, which may be applied to another class of diseases, not inflammatory. We have, in this sense, strabismus, laryngismus, pharyngismus, cheirismus, podismus, &c.

The term *trachelismus* may be used to express that paroxysmal affection of the neck, in which, the muscles acting inordinately, the neck is affected with opisthotonos, or becomes twisted, or otherwise contorted; whilst the subjacent veins are subjected to compression, and the blood flowing along them is arrested or impeded in its course—a condition which may be aptly termed *phlebismus*. The term phlebismus may be regarded as generic, and each kind of this affection may have its appropriate and specific designation; and whilst the term *sphagiasmus* denotes compression of the internal jugular vein, that of *rhachiasmus* may be used to denote that scarcely less important event of interrupted circulation in the rachidian or vertebral vein. The former will be henceforth associated with paroxysmal apoplexy, paralysis, mania, &c.; the latter with the epileptic seizure, and other convulsive and spasmodic affections, the cerebrum and the medulla oblongata being affected respectively.

Trachelismus and phlebismus constitute one of the most important events in *pathology*, and especially in the pathology of the nervous system. Induced by mental emotion and excitants of reflex action, they are, in their turn, the fruitful source of *congestion* in the *cerebrum*, or in the *medulla oblongata*, and of *cerebral* and *spinal paroxysmal diseases*.

There is no *order*, no *degree*, in which the muscles of the neck may not act, and in which the veins of the neck may not be compressed; there is no form of cerebral and spinal paroxysmal derangement—from a momentary oblivium or delirium to coma or mania—from the slightest spasmodic or paralytic affection to epilepsy or hemiplegia—which may not take place as consequences of that compression. Having the limits of these maladies clearly placed before our eyes, we may readily imagine or comprehend the intermediate forms, mild, and dire, and we are prepared for their immediate observation.

To trace these maladies from their faintest to their darkest shade—to trace them back to their causes, moral and physical, and onwards, to their dire effects on the intellect and on the limbs, of excitement or of stupor, of spasm or of paralysis, through trachelismus and phlebismus—is to engage in the investigation of one of the most novel, varied, and practically useful subjects in the domains of pathology. From mere sick-headache, to paroxysmal apoplexy, or epilepsy, this *Class* of diseases extends, occupying with its varied forms the lengthened interval.

A remarkable confirmation of these views is afforded by the phenomena presented by *Strangulation*. The moment the cord is tightened round the neck, *apoplectic* insensibility takes place; as a subsequent phenomenon, we have *epilepsy*, the tongue is protruded and bitten in some cases, and there are erection and seminal emission in others; *asphyxia* terminates the dreadful series of events. It will be plain, from what has been said, that we cannot concur with the eminent physician of Edinburgh, that "strangulation, when the neck is not disclocated, appears to be simple apoplexy."

In slighter degrees of strangulation there are slighter effects, but amongst these there is always insensibility. In one case, a youth, on the eve of an execution, impressed with the terror of the coming scene, wished to experience the sensation induced by strangulation; and having so arranged a cord on a horizontal pole (the upper bar of a stile crossing a village foot-path) that it hung down loosely, he laid his neck gently upon it. Insensibility was produced, and the poor youth was found dead, having committed an undesigned suicide. The coroner for Middlesex has had frequent opportunities of observing the real suicide strangled in such a position that the slightest pain or irresolution might have led him to rescue himself had consciousness remained.

We remember the sad fate of the American diver, when he attempted to suspend himself, apparently by the neck, on Waterloo Bridge. The cord slipped, and really tightened round the neck: no attempt to save himself was made—no moment of consciousness remained.

A boy mentioned by Zitzilius, as quoted by Abercrombie, had drawn his neckcloth remarkably tight, and was whipping his top, stooping and rising alternately, when, after a short time, he fell down apoplectic. The neckcloth being unloosed, and blood being drawn from the jugular vein, he speedily recovered.

We are all acquainted, too, with the recorded phenomena of Thuggee: the kerchief is tightened, and consciousness, and every mental and physical power, are extinct in a moment. Similar effects have been observed in animals. The first Monro suspended a dog by a cord, excluding the trachea: insensibility was produced, but not asphyxia.

I was once witness to an experiment of Sir Astley Cooper. He compressed the carotid and vertebral arteries, as he thought, in a rabbit, with the thumb and fingers; I believe it was the jugular and vertebral veins; insensibility and convulsions ensued, and ceased with the compression of the veins.

There is another fact of the same kind. The horse is subject, when drawing against the collar, to what is termed the megrims, (hemicrania.) In one such case witnessed by my friend Mr. H. Smith, the external jugular vein, having been previously opened, burst out bleeding.

A more distinct experiment of Sir Astley Cooper is the following:—

"In one rabbit I tied the jugular veins on each side of the neck. When it was set at liberty, it ran about, cleaned its face with its paws, and took green food.

"Its respiration was reduced to 68 inspirations in a minute, which is about half the natural number. After four hours, it ran about as if nothing had happened, and eventually recovered.

"When it was killed and injected, I found on each side, three anastomosing veins passing from the anterior to the posterior part of the jugular veins, and conveying the blood from the head to the heart; but the vertebral vein had remained whole, and become enlarged; and it passed on the fore-part of the vertebræ, from the head to the space between the fourth and fifth cervical vertebræ, where it entered the vertebral canal.

"In a second rabbit I tied the jugular veins on each side of the neck, as before. The animal's respiration became slow; but it ate green food, ran about, and was difficult to catch; but for five days after it appeared dull; its ears had dropped. On the seventh day, it was seen to be convulsed, and frequently rolled over. Its voluntary powers were lost, as well as the sensation, in a great degree. On this day it died. On examination a clot of blood was found extravasated in the left ventricle of the brain.

"Hence, it follows, that apoplexy will occasionally result from an obstruction to the return of blood in the jugular vein; and this I have known to happen from enlargement of the glands in the neck of a boy."

To these observations I may add the following interesting extract from Abercrombie, to show at once the importance and the obscurity of the subject, previous to these inquiries. After having spoken of the effects of strangulation, and of the "numerous examples in which persons fall down suddenly in a state of perfect apoplexy, and very speedily recover under the appropriate treatment, without retaining a trace of so formidable a malady," he adds—

"The apoplectic attack, as it occurs in such examples as these, must be supposed to depend upon a cause which acts simply upon the circulating system of the brain, producing there a derangement which takes place speedily, and is often almost as speedily removed. *What the precise nature of that derangement may be, is a point of the utmost difficulty to determine.*" Abercrombie's philosophic mind felt, in all its force, the want of some such principle as it is the object of these observations to set forth.

I may now repeat the phenomenon of *blushing* is familiar to every one. Obviously the effect of emotion, I have suggested the contraction of the platysma myoides on the external jugular vein as the second link of the chain of causes and effects, and the impeded return of blood along this latter, as the third.

Let us imagine a similar condition of the *internal* jugular; there will be a state of *blushing*, in other words, of congestion of the cerebrum, with oblivium, stupor, or even apoplexy. Or let the vertebral vein be so affected, and a similar condition of *blushing* of the medulla oblongata will accrue, with varied spasmodic affections, such as strabismus, laryngismus, or even epilepsy. I have actually seen a state of recurrent *venous blushing* of the hand of an infant affected with convulsion.

If these veins be so affected, not singly, but conjointly, it is obvious that a more complicated result will take place; and thus venous lividity of the face and hands, is apt to be conjoined with the apoplectic and epileptic states, and these with each other.

If one part, or hemisphere, of the cerebrum be more affected than the other, we may witness a form of paralysis, which, as it usually ceases with the congestion, I have designated *paroxysmal*.

Or another *degree* or *locality* of the *blushing* of the cerebrum may lead to a paroxysm of mania.

A momentary unconsciousness,—cerebral epilepsy,—occurs from modes of strangulation such as I have described as constituting trachelismus. I will only *allude* to the peculiar effect said to be produced on the sexual system,—spinal epilepsy,—by slighter degrees of strangulation.

A slight degree of trachelismus leads to the feeling of "strangulation," or of "constriction," or of "tightness," or "pressure," or "fulness," about the neck; for these and other phrases are used by patients.

In inquiring into these feelings, we must avoid leading questions, and make the patient express himself. "Do you experience anything unusual about this part?" (referring to the front of the neck generally,) is the only question I ever allow myself to ask. The reply to this is frequently of the most unexpected and interesting kind.

The subjects of trachelismus and phlebismus bring under our review the cases of paroxysmal apoplexy, paralysis, mania, and epilepsy, in all their shadowy and terrific forms. In their causes, their nature, their effects, and their treatment, they present to the physician a field of investigation, equally uncultivated hitherto, and prolific in results.

A chief cause is *emotion.* Such was the cause of the epileptic attack experienced by Henry Kirke White; and in general, I believe the mental agitation, the hopes and fears attendant on the examinations for honors at Cambridge, far more dangerous than the intellectual efforts previously made; and I would strongly and loudly urge on the Senate of that famed University (and others) some other, and better, and more continuous mode of judging, than the *one* fearful and dangerous trial now adopted.

A volume—and an instructive volume—might be written on the dire effects of the emotions and passions, in their varied kinds, in inducing trachelismus and phlebismus, and their results. Next should be investigated the effects of sexual excesses. And lastly, the effects of the excitants of spinal action, reflex and direct, should be traced from link to link through the fearful chain.

All these effects must be carefully distinguished from those of inflammation and of organic lesion, and the task of an accurate *Diagnosis* is not an easy one.

In my former paper, I observed, "It may be laid down as a principle, that there is no muscle—no set of muscles—in the neck, which may not become spasmodically contracted, singly, or conjointly with others; and that there is no vein in this region which may not, under the influence of such contraction of muscles, become compressed, and the course of whose blood may not consequently become impeded." "As a further consequence, there is no organ which delivers up its blood to such vein, which may not be the seat of congestion, and, if I may so express myself, of the apoplectic state." As a still further consequence, I may now add, that there is no paroxysmal affection of the nervous system, and especially of its *cerebral* and its *spinal* portions, however severe, however apparently slight, which may not result from this series of causes and effects.

This statement may be expanded into the different modes and forms of diseases of the nervous system, in their cerebral and spinal portions.

Hence we have stupor, oblivium, cerebral epilepsy, vertigo, headache, delirium, flashes of light, muscæ, flocci, dimness, amaurosis in every degree, tinnitus, and other noises, dulness, deafness ; neuralgia and other morbid sensations ; paralytic affections ; hence we have epilepsy, and every kind, mode, and degree of spasmodic and convulsive affection.

So far the efforts of *trachelismus* are *paroxysmal ;* they supervene on emotion or harass of mind, and on excited reflex action, and recede completely. But there is a limit to this complete recession. At length the recession is incomplete. The effects of the congestive state of the veins are more or less permanent, in the form, generally, of slight paralysis. What is the precise condition of the intermediate blood-channels ? and of the minute arterial branches and venous roots ? Do they yield and dilate, as we sometimes see minute ecchymoses on the face ? Is their lesion of their intimate tissue ? Is there effusion ? Is there ramollissement of the adjacent cerebral or spinal tissue ?

In a word, it has been admitted, from time immemorial, that mental agitation and passion, and deranged condition of the stomach, liver, bowels, &c., induce apoplectic attacks. I only attempt, for the first time, I believe, to set forth the *rationale* of this pathological phenomenon.

In doing this I establish a *Class* of *paroxysmal* diseases of the nervous system, cerebral as well as spinal ; apoplectic and paralytic as well as epileptic and convulsive. I endeavor to fix the attention of physicians, and of patients too, on this, the *curable* stage of these dire maladies, before, from being merely paroxysmal derangement, they pass into permanent lesion of tissue.

This is *living* pathology as contrasted with the results of morbid actions as usually detailed in books, which are *dead.* If we read over the titles of each of the nine *Letters* of M. Lallemand, we find but a list of the capita mortua of the real and living disease. But what actions led to each of these ? This is *the* one question, with those of the diagnosis and of the treatment, which interests the physician and the patient.

Inflammation and its causes ; venous congestion and its causes ; certain conditions of the vascular system or its contents—as anæmia, plethora, the effects of alcohol ; the tuberculous diathesis, diabetes, albuminuria, a syphilitic taint, &c.; the seat of the disease in the membranes, in the substance of the cerebrum and spinal marrow ; the character of the disease, especially as to its acute or chronic form, &c. I mention these circumstances to show how extensive a question that of the diagnosis is.

To return to the subject of trachelismus and of phlebismus, and of the paroxysmal affections of the nervous system, their effects, I must repeat that they exist in *two* stages ; the first, that in which none but a functional change has taken place ; the second, that in which physical lesion has supervened. The former case is the truly paroxysmal. These two conditions are coincident and commensurate with each other. As lesion supervenes, the symptomatic phenomena becomes more or less persistent ; and here again the phenomena are proportionate to each other. In the severest form of this affection, physical lesion, perhaps rupture, may occur at once. It may be apoplexy or hemiplegia.

It would be interesting to institute a comparative estimate of the importance of inflammation and of the congestive state to which I have adverted, in regard to the nervous system. I believe the latter to be the most frequent cause of disease of the *substance* of the nervous centres, and its effects to be frequently mistaken for those of inflammation, which is the most frequent, but not the sole cause of affection of the *membranes*, chiefly arachnitis. This statement must be left to future observation for development. The idea is big with conclusions of the deepest interest.

The specific effects of impeded venous circulation, whenever this occurs in external parts, are sure to be *two;*—first, dilatation, or the *varicose* state; and secondly, *effusion of serum.* We may suppose, until further investigation has confirmed or corrected this view, that similar events occur in the nervous centres, leading to irritation or lesion of their structure. Rupture, or softening of their substance, may follow if the proper remedies be neglected.

I now proceed to discuss briefly the subject of the treatment.

The causes—all mental emotion, agitation, passion, must be most carefully avoided; all sources of gastric and intestinal irritation must be removed.

I believe the most specific preventive to be systematic walking exercise, and especially a pedestrian tour.

I am convinced that I have seen the best effects from a light mercurial conjoined with ipecacuan and squill, and an aperient, taken so as gently to effect the mouth and act on *all* the secretions and excretions, the mercurial cachexia being prevented by air and exercise.

The head should be kept cool by a lotion, consisting of one part of alcohol and three of water, the feet guardedly warm and dry.

Sinapisms should be applied to the nape of the neck, extending to behind the ears. Dry cupping, and cupping with simple or crossed incisions, and with the detraction of the appropriate quantity of blood, have proved most useful.

But this is rather the treatment of the permanent effects of congestion. The most important treatment consists in avoiding the exciting causes of trachelismus, and its effects. It is obvious that, if we would save the brain, and save the intellect and limbs, we must deplete the veins?

We may now resume our subject, and observe that what shame does to the face, neck, and breast, through the contraction of the platysma myoides on the external jugular, other agitations and passions, and excitants of reflex action, effect on the cerebrum and medulla oblongata, or both, by the compression or obstruction of the internal jugular or vertebral. The former is *seen*, the other is *deduced* from the symptoms, consisting of paroxysmal, cerebral or spinal seizures. If repeated, the cerebral or spinal veins, or the intermediate blood channels between these and the arteries, become dilated or varicose, or yield an effusion of serum, whence persistent forms of the same diseases. Of these effects, the first should be subdued, the second should be averted, by timeous, that is, prompt, and effectual *cupping*.

In conclusion, I beg to observe, that I have not in these observations even attempted to avoid the repetitions which my reader will doubtless observe. I believed the importance and novelty of the subject justified them. In a THIRD SERIES I propose to treat chiefly of a parallel between *Inflammation* and *Congestion* of the Nervous Centres.—*London Lancet.*

